# Developing Small Molecule Therapeutics for the Initial and Adjunctive Treatment of Snakebite

**DOI:** 10.1155/2018/4320175

**Published:** 2018-07-30

**Authors:** Tommaso C. Bulfone, Stephen P. Samuel, Philip E. Bickler, Matthew R. Lewin

**Affiliations:** ^1^California Academy of Sciences, San Francisco, 94118 CA, USA; ^2^Ophirex, Inc., Corte Madera, 94925 CA, USA; ^3^University of California, San Francisco, 94118 CA, USA; ^4^General Medicine, Queen Elizabeth Hospital, King's Lynn, PE30 4ET, Norfolk, UK

## Abstract

The World Health Organization (WHO) recently added snakebite envenoming to the priority list of Neglected Tropical Diseases (NTD). It is thought that ~75% of mortality following snakebite occurs outside the hospital setting, making the temporal gap between a bite and antivenom administration a major therapeutic challenge. Small molecule therapeutics (SMTs) have been proposed as potential prereferral treatments for snakebite to help address this gap. Herein, we discuss the characteristics, potential uses, and development of SMTs as potential treatments for snakebite envenomation. We focus on SMTs that are secretory phospholipase A2 (sPLA_2_) inhibitors with brief exploration of other potential drug targets on venom molecules.

## 1. Introduction

Snakebite envenomation is a neglected tropical disease that causes more than 100,000 deaths every year [1, 2]. Of the snakebites that are ultimately fatal, it is estimated that about 50-75% occur before victims can reach the hospital for antivenom treatment [3–6]. There is an urgent need for novel interventions to address the therapeutic and temporal gap between a bite and hospital-level care. Small molecule therapeutics (SMTs) have been proposed for initiating the treatment of snakebite in the prehospital environment and as adjuncts to antivenom therapy [7, 8].

## 2. Small Molecule Therapeutics

**Figure 1:**
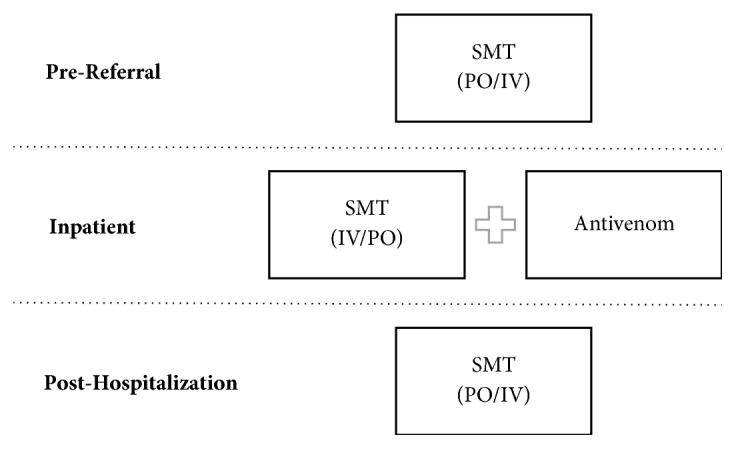
Potential uses of an SMT, via PO (oral), or IV (intravenous).

SMTs represent a potentially useful adjunctive therapy to antivenoms, the current mainstay of care for symptomatic snakebite. As a group, most SMTs are naturally occurring (*e.g*., alkaloid) or synthetic molecules that are usually intended to act on specific targets. G-protein coupled receptor systems comprise the largest group of targets for SMTs [9, 10]. SMTs could be used in multiple ways to decrease morbidity and mortality caused by snake envenomation (Figure 1). Ideally, an SMT could be given orally in the prereferral setting to diminish or delay venom toxicity. An SMT could also be used in an in-patient setting, either orally or intravenously, as an adjunct to antivenom and to increase the breadth of treatment efficacy. These uses could potentially reduce the required dosage of antivenom and improve treatment costs by improving the performance of imperfectly matched antivenoms. Finally, an SMT could be administered posthospitalization to reduce the chances of rebound effects from venom components not effectively or durably covered by antivenom. With low toxicity and high efficacy, SMTs could even be considered for prophylactic use in high-envenomation risk situations.

The search for nonserotherapy antidotes to snakebite is not new. Traditional healers have long used poultices and teas derived from plants to attempt treatment. Plants and fungi remain the basis for most active pharmaceutical ingredients used in modern medicine today [11]. The spectrum of small molecule inhibitor compounds for the treatment of snakebite has been reviewed by Carvalho, Soares, Laustsen, Bastos, and others [7, 12–16]. As a field, only a small number of individuals and groups have directly addressed the question of developing small molecules for the treatment of snakebite and it remains largely unexplored [8, 17–20]. No SMT for snakebite treatment has ever been approved for use in humans or animals. The potential benefits of using SMTs as an adjunctive therapy deserve further study.

**Figure 2:**
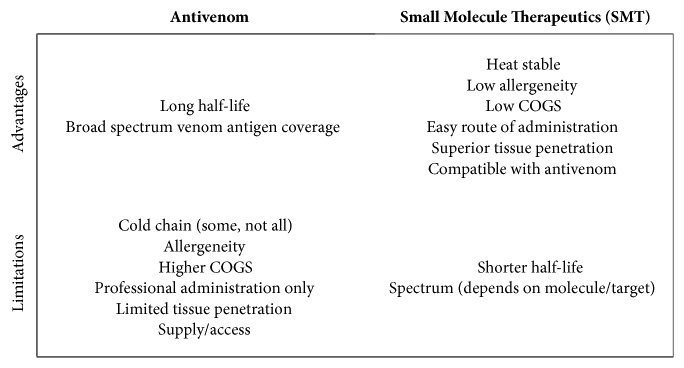
Advantages and limitations of antivenom and SMTs. If proven effective, an SMT might address some limitations of antivenom and vice versa. (COGS = Cost of Goods).

SMTs have many characteristics that make them potentially useful as an adjunctive therapy for snakebite treatment. If proven effective, SMTs might address some of the most significant limitations of antivenom (Figure 2) [21]. By their nature, SMTs are at low risk for allergenicity or anaphylactic shock as compared to most serum-based therapies [22]. In addition, many snake venoms have in common active toxic components that could be targeted by SMTs, including the secreted phospholipase A2 (sPLA_2_) and metallo- and serine-proteases (svMPs and SPs, respectively). If the inhibitory targets are common among snake species, SMTs could potentially have broad spectrum “venom agnostic” effects. This would potentially decrease the importance of snake identification and would increase the usefulness of SMTs as first-line therapeutics. Venom agnostic SMTs could be used in a broad range of geographical areas and potentially eliminate the need of an expert to confirm snake species prior to initializing treatment, though newly developed rapid diagnostics could rapidly refine the specificity of treatment and improve the clarity and powering of clinical studies where more than one type of venomous snake is prevalent [23, 24]. Finally, in theory, multiple SMTs developed against various venom proteins could be combined to inhibit wider varieties of toxins present across snake species.

For SMTs to be potentially useful as an adjunctive therapy for snakebite, they should be heat stable and easily administered, allowing point-of-care treatments in the field. Also, the manufacturing cost-of-goods (COGS) of SMT should be comparatively low. Consideration of repositioned (repurposed) compounds with a history of use in humans, a strategy discussed in detail below, could further decrease costs of development [8, 25]. Also, venom distributes outside the blood, with an average volume of distribution in animals of 0.054-0.070 L/Kg, where antivenom cannot distribute [26]. Given their small molecular weight and charge, SMTs will generally have much higher volumes of distribution and tissue penetration than antivenom, allowing them to distribute within vulnerable tissues [26–30]. The addition of tissue penetrating molecules could expand the time during which severe neurotoxicity could be reversed. This would be an advance in the field because of antivenom's limited access to synaptic junctions and inability to reverse critical impairments such as respiratory paralysis.

WHO has provided a detailed preclinical assessment for antivenom development for snakebite treatment and has recently been reviewed in detail by Gutierrez et al. [31, 32]. However, none exists for SMT development for snakebite. Based on the WHO guidelines, previous studies on antivenom development, and our experience in the development of SMTs, the preclinical assessment of SMTs for snakebite treatment can be envisioned (Table 1).

## 3. Pathway for Development of an SMT

### 3.1. Venom Target Selection

A toxin-centric approach might be the basis of next-generation snakebite treatment [33]. As described by Laustsen, snake venom is likely to be the “most complex pharmaceutical target” known, composed of a multitude of toxin components and complex biochemical interactions [34]. Thus, venom targets for inhibition should be, ideally, abundant across as many of the medically important snake species as possible. For understanding venom properties and targeting, proteomic analysis of snake venom has been crucial to reveal species variation in venom composition and toxicity [35–37]. Proteomic analysis has revealed a wide array of active toxic ingredients from at least 26 protein families, but the most common medically relevant components are found within four families in varying proportions [38–40]. These proteins are secreted phospholipase A2 (sPLA_2_), metallo- and serine-proteases (svMP and SP), and the nonenzymatic three-finger toxins (3-FTX) [35, 39–41]. Not all snake venoms, however, have unique toxins that are in this group of four, including mambas with dendrotoxins and some rattlesnakes with low molecular mass cationic myotoxins [42–44]. Continued research into the proteomic and toxicovenomic characterization of the most medically relevant venoms is crucial in order to have a more comprehensive understanding of drug and antivenom targeting in these species, as well as to understand the nature of therapeutic failures when they occur. The use of tools, such as the newly developed Toxicity Score, which combines the medical importance and the relative abundance of a specific toxin, can aid the identification of a target [34, 35].

Because of its ubiquity and clinically significant effects, we focus on sPLA_2_ as a candidate for inhibition by SMTs [31, 40]. Snake venom sPLA_2_ play roles in early- and late-onset symptomology, as well as synergistic and regulatory roles for other coexisting snake venom components [45–52]. sPLA_2_ are also some of the most pharmacologically active, multieffect (neuro-myo-cyto-hemotoxic) venom components (Table 2) [45–53].

svMPs represent another important target for inhibition because of their systemic and local toxicity, caused by fibrinolytic and hemorrhagic activity among others [1, 49]. Recent studies suggest that small molecules, in particular anticancer metalloprotease inhibitors and, possibly, metal chelators related to EDTA, have inhibitory effects on svMPs [18, 54–57]. SP inhibitors will be amongst the most challenging to develop because of their complex role in coagulation and short half-life of molecules that have made it to clinical use thus far, such as gabexate [58]. The most active work in this area has come from Vaiyapuri [59, 60]. 3-FTX toxin lacks enzymatic activity and presents a challenging target for an SMT. In addition, there is potential for single toxin inhibitors to affect synergistic effects of toxins, for example, sPLA_2_ potentiation of svMP in* Bothrops alternatus* venom [34, 61].

**Figure 3:**
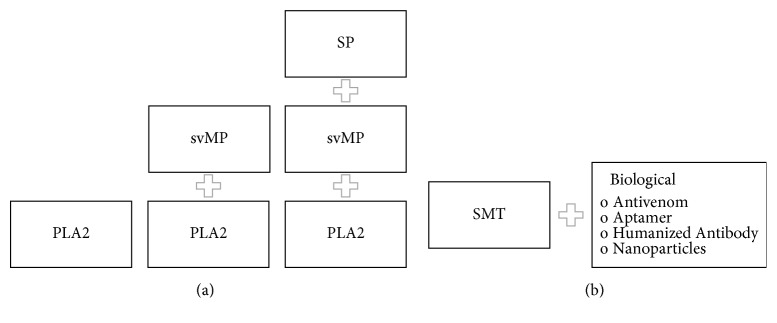
Hypothetical pipeline of SMTs for snakebite treatment. (**a**) Targeted inhibition of major snake venom enzymatic toxins, secreted phospholipase A2 (sPLA_2_), and metallo- and serine-proteases (svMP and SP), through a combination of multiple inhibitory small molecules. (**b**) In combination with biologicals or others as adjuncts to antivenom for hospital administration (e.g., for targeting non-enzymatic toxins, such as 3-FTX).

While sPLA_2_ inhibition might prove sufficient as a “bridge-to-survival” for many types of venoms when administered in a prereferral setting and, at times, be sufficient for treatment, future SMTs might be mixtures of other SMTs (Figure 3(a)). Some targets could also be inhibited indirectly by SMTs, such as 3-FTX, whose effects might sometimes be mitigated by acetylcholinesterase inhibitors, though the use of these inhibitors remains controversial despite decades of use for this purpose [41, 50, 62–64]. In addition, SMTs might be used to slow the spread of venom by paralyzing lymphatic smooth muscles (e.g., with lidocaine) [65]. SMTs could also be paired with antibodies or other biologicals to increase the range of efficacy or extend their paraspecificity (Figure 3(b)).

### 3.2. Strategies for Discovery of Lead Compounds

**Figure 4:**
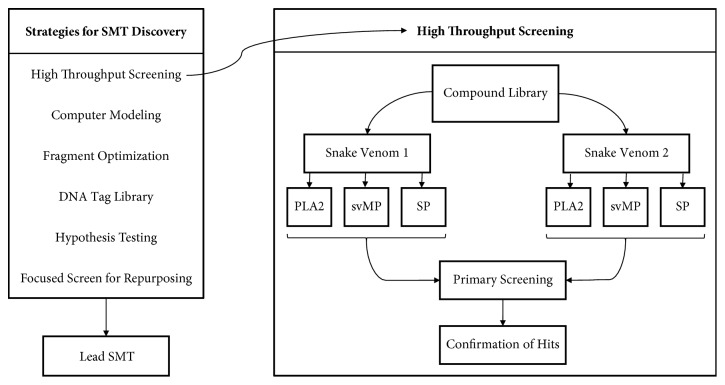
Hit to Lead: a variety of strategies to discover new SMTs and an example of processes and targets for High Throughput Screening (HTS) of candidate snake venom SMTs. sPLA_2_, svMP, and SP serve as examples of potential targets. Different assay methods are used for each type of enzymatic activity so screens would be run separately even if compound libraries were the same.

Several strategies to discover new SMTs are commonly used and illustrated in Figure 4. Some strategies involve the screening of entire compound libraries against the selected target, such as High Throughput Screening (HTS). HTS requires no previous knowledge of potentially successful chemotypes, but it does require a venom-relevant assay. Compounds that show a predetermined percentage of inhibition, for example, more than 50% inhibition at 10*μ*M, are advanced to the Confirmation of Hits stage. Confirmation of Hits would test the screened compounds with a dose-response curve, utilizing multiple concentrations, to determine the IC_50_ (half-maximal Inhibitory Concentration) and, therefore, the effectiveness of the compound at inhibiting the active components of snake venom. Other methods, such as focused screening, are less time consuming but require more knowledge. Focused screening involves screening a small amount of existing developed drugs for potential repurposing for snakebite. While the strategy chosen to discover a lead snakebite SMT depends on the resources and knowledge available to the investigator, it is important that the correct assay for screening is used. If the assay does not reflect the relevant venom toxicity, it could be either worthless or a very useful molecule could remain undiscovered.

### 3.3. Repurposing as a Strategy for Discovery and Development

**Figure 5:**
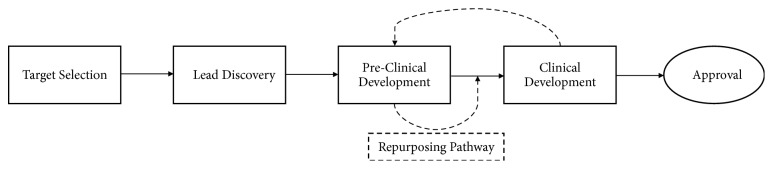
Scheme of a potential SMT development pathway. “Sections” correspond to paragraphs that follow. The repurposing pathway accelerates development and lowers costs by starting at a more advanced stage of development than a new chemical entity.

To achieve a lower-cost SMT product that can be commercialized and priced sustainably, its development costs need to be lowered. The development of a new drug from lead discovery to launch can take many years and cost more than one billion USD [66]. Repurposing, a strategy for accelerated drug development by reviving or expanding indications of existing drugs, might be beneficial to the development of a potential SMT for snakebite. Repurposing, or repositioning, is a powerful way to reduce the cost of drug development, particularly for neglected tropical diseases that do not offer sufficiently alluring markets to larger pharmaceutical companies, such as snakebite [25, 67]. Repurposing compounds already in development can accelerate entry to clinical trials and result in significant savings. Repurposing can also revive the potential of drugs that never reached commercialization or expand the purpose of existing drugs by applying them to new indications [68]. Examples of successfully repurposed drugs include Thalomid (Thalidomide) for treatment of leprosy and Viagra (Sildenafil) for pulmonary hypertension [25, 68]. In 1972, Banerjee et al. presented an early example of repurposing an SMT for snakebite when neostigmine was used to treat the paralytic effects of an elapid bite [41]. Multiple groups have since investigated the use of this class of acetylcholinesterase inhibitors in the clinical or laboratory setting with variable results [41, 50, 63, 64]. Development of a hypothetical SMT using a repurposing pathway is shown in Figure 5.

Drug repurposing does not generally require further optimization or structural modification of an FDA-approved drug or lead compound, unless formulations were altered from original studies, which may require extra characterization and testing [25]. Therefore, data from efficacy, safety, pharmacokinetics/dynamics studies, and others conducted for the initial indication of the compound can be reused for the new indication if the originators donate, license, or sell access to their data [69]. In regards to safety, Klug et al. note that “the most common side effects of the repurposed drugs are minor in comparison to those of many existing NTD therapeutics” [25]. Repurposing can be a cost-effective, lower-risk strategy to rapidly develop new SMTs for snakebite treatment.

## 4. Repurposed Drugs and Model SMT Candidates for Enzymatic Inhibition of Snake Venom

We recently identified a previously studied sPLA_2_ inhibitor, varespladib (*syn* LY315920, S-920), and its orally bioavailable prodrug, methyl-varespladib (*syn* LY333013, A-002), as a candidate treatment for snake envenomation [8]. Varespladib appears to be a potent sPLA_2_ inhibitor against a broad spectrum of snake venom sPLA_2_s. As mentioned above, the ubiquity and clinically significant effects of snake venom sPLA_2_s across venom types make it a plausible candidate for inhibition by an SMT with potential for broad spectrum of efficacy.

Varespladib, for the indication snakebite, is an example of a potentially repurposed compound. In recent decades, several large pharmaceutical industry endeavors focused on sPLA_2_ inhibition for potential anti-inflammatory and cardiovascular drugs but, to date, none came to market [70, 71]. sPLA_2_ enzymes are present throughout the animal kingdom and are involved in multiple key processes, such as synaptic transmission and inflammation. sPLA_2_ are implicated as having roles in various important diseases such as sepsis, cardiovascular disease, neurological disease, rheumatological disease, and cancer [72]. In attempts to treat these diseases, several companies devoted extensive resources to developing sPLA_2_ inhibitors as targets for drug development. These inhibitors could be considered for repurposing as SMTs for snakebite treatment.

**Figure 6:**
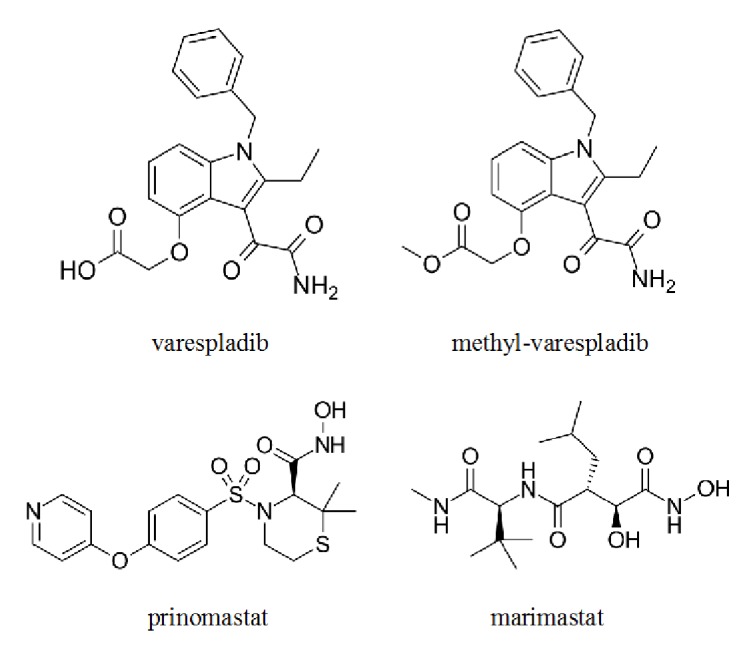
Structure of candidate SMTs for repurposing: varespladib (top left), its orally bioavailable pro-drug, methyl-varespladib (top right), prinomastat (bottom left), and marimatsat (bottom right). Marimastat and prinomastat are both orally bioavailable and could be combined (mixed or copackaged) for more extensive coverage as field antidotes [1, 54].

Figure 6 shows the structures of the sPLA_2_ inhibitor, varespladib, and other SMT candidates that could be repurposed, the anti-svMP peptidomimetic SMTs, prinomastat, and marimastat [17, 18, 54]. Varespladib is a sPLA_2_ inhibitor originally developed by Shionogi and Lilly, for treatment of pancreatitis and sepsis and, later, licensed to Anthera, for treatment of acute chest syndrome and heart disease [73, 74]. This makes it an inviting candidate for repurposing because of the known safety profile and, thus, potentially reduced development costs [8, 75, 76]. Similarly, MP inhibitors that have previously been developed for cancer treatment such as batimastat, marimastat, and prinomostat could also be considered [11, 12, 77, 78].

*In vitro*, varespladib was observed to be a surprisingly potent inhibitor of snake venom sPLA_2_. The observed consistent potency (nano- and subnanomolar range) against a range of sPLA_2_ from more than 25 medically important snakes from six continents suggests that these could be scaled for human use at reasonable dose volumes and dosage forms [8].* In vivo*, rescue studies using lethal doses of coral snake (*M. fulvius*) and common adder (*V. berus*) venom were performed on mice to whom venom was administered subcutaneously followed by intravenous varespladib in the lateral tail vein, and all survived for at least 24 h, while those receiving only venom died in a matter of minutes or hours. Similarly, mice subjected to intraperitoneal administration of venoms were rescued by intravenous, intramuscular, and oral routes of drug administration against venoms from snakes such as* D. russelli*,* E. carinatus sochureki*,* O. scutellatus*,* C. scutulatus*, and* C. durissus terrificus* (unpublished data). In addition, recent results by Wang et al. (2018) showed the inhibitory effect of varespladib treatment on* D. acutus, A. halys, N. atra*, and* B. multicinctus in vitro *and* in vivo* [79]. The results of these experiments have led to several new questions, including the exact mechanism by which survival is enhanced by these experimental drugs.

For a repurposed compound, attention should be given to the safety signals seen in prior trials. Consideration of the difference in use of an SMT between previous indication and snakebite might mitigate safety risks: for example, an SMT for snakebite would be used acutely, with one or few doses, rather than chronically. If use of SMTs includes early administration in the field, potentially before appearance of signs of envenomation, safety of the drug for individuals bit by nonvenomous snakes or individuals exposed to “dry” bites must be considered. In terms of efficacy, the risk that inhibition of one toxin (e.g., sPLA_2_ or svMP) might not be sufficient and can be mitigated by addressing additional targets and having backup molecules potentially more suitable for different geographic regions [54]. Lastly, means for commercialization should be evaluated early on to determine real world feasibility of implementing an SMT.

## 5. Conclusions

SMTs are potentially useful tools and could meet key development criteria as initial field treatment for snakebite and as adjuncts to antivenom with unrealized potential.

The rational development of an SMT commences with selecting an inhibitory target and determination of the breadth of efficacy across snake species. The breadth of effect helps determine the applicability to specific geographical regions and snake types. Aiding the discovery step is the availability of SMTs already developed by the pharmaceutical industry for other indications, which, if repurposed, could substantially lower development costs and increase the potential for accelerated approvals. Preclinical studies to evaluate the safety and efficacy of the SMT follow similar assays used in antivenom testing, but other assays to test heat stability, ease of administration, and bioavailability are likely to be additionally performed. A careful, systematic, and multidisciplinary approach will be required to determine the most appropriate next steps in the development and deployment of new therapeutic classes for the initial treatment and overall management of snakebite.

## Figures and Tables

**Table 1:** Desirable preclinical characteristics of an SMT for snakebite.

**Safe**	(i) Minimize off-target toxicity [66]
	(ii) Without adverse interactions with antivenom
	(iii) Broad therapeutic index

**Efficacy *(in vitro)***	(i) Nano- or subsnanomolar *in vitro* potency (IC_50_) for scalability [66, 77, 80]
	(ii) Determination of affinity, minimum active concentrations, physical characteristics, stability, mechanisms of action, dose-response, and drug effects [8, 31, 32, 54, 69, 77, 80–87]

**Efficacy *(in vivo)***	(i) Tested with both:
	(a) Minimum acceptable: Pre-mixing of venom and antidote prior to injection (ED_50_ determination) [32]
	(b) Ideal: Venom administration prior to administration of antidote [8, 31, 88]
	(ii) Compatible with standard assessments of coagulation [89]

**Broad Spectrum**	(i) Wide target selection (ubiquity and medical importance of inhibitory target amon snake species) [34, 35]

**Heat Stable**	(i) Real-time stability studies up to 37°C (±2°C) and relative humidity of 75% (±5%) (WHO “Climatic Zone IVb”) [90]

**Ease of Administration**	(i) Oral solution, rectal or nasal formulations
	(ii) Auto-Injectable [54]

**Bioavailability**	(i) For oral formulations, adequate bioavailability in fed state

**Half-life**	(i) For field antidotes, half-life of at least 5 to 7 hours [78, 91, 92]
	(ii) Potential for re-dosing

**Table 2:** Generic pathogenesis of major toxins in snake venom: secreted phospholipase A2 (sPLA_2_), metallo- and serine-proteases (svMP and SP), and the nonenzymatic three-finger toxins (3-FTX).

**Family**	**sPLA**_**2**_	**svMP**	**SP**	**3-FTX**
Neurotoxic	**Yes**	-	-	Yes
Hemotoxic	**Yes**	Yes	Yes	-
Myotoxic	**Yes**	Yes	-	-
Cytotoxic	**Yes**	Yes	-	Yes
